# Erosion protection benefits of stabilized SnF_2_ dentifrice versus an arginine–sodium monofluorophosphate dentifrice: results from in vitro and in situ clinical studies

**DOI:** 10.1007/s00784-016-1905-1

**Published:** 2016-08-01

**Authors:** N. X. West, T. He, E. L. Macdonald, J. Seong, N. Hellin, M. L. Barker, S. L. Eversole

**Affiliations:** 1 School of Oral and Dental Sciences, Bristol Dental School and Hospital, Lower Maudlin Street, Bristol, BS1 2LY UK; 2 Procter and Gamble, Cincinnati, OH USA

**Keywords:** Erosion, Tooth wear, Dentifrice, Clinical trial

## Abstract

**Objectives:**

The aim of these investigations was to assess the ability of two fluoride dentifrices to protect against the initiation and progression of dental erosion using a predictive in vitro erosion cycling model and a human in situ erosion prevention clinical trial for verification of effectiveness.

**Materials and methods:**

A stabilized stannous fluoride (SnF_2_) dentifrice (0.454 % SnF_2_ + 0.077 % sodium fluoride [NaF]; total F = 1450 ppm F) [dentifrice A] and a sodium monofluorophosphate [SMFP]/arginine dentifrice (1.1 % SMFP + 1.5 % arginine; total F = 1450 ppm F) [dentifrice B] were tested in a 5-day in vitro erosion cycling model and a 10-day randomized, controlled, double-blind, two-treatment, four-period crossover in situ clinical trial. In each study, human enamel specimens were exposed to repetitive product treatments using a standardized dilution of test products followed by erosive acid challenges in a systematic fashion.

**Results:**

Both studies demonstrated statistically significant differences between the two products, with dentifrice A providing significantly better enamel protection in each study. In vitro, dentifrice A provided a 75.8 % benefit over dentifrice B (*p* < 0.05, ANOVA), while after 10 days in the in situ model, dentifrice A provided 93.9 % greater protection versus dentifrice B (*p* < 0.0001, general linear mixed model).

**Conclusion:**

These results support the superiority of stabilized SnF_2_ dentifrices for protecting human teeth against the initiation and progression of dental erosion.

**Clinical relevance:**

Stabilized SnF_2_ dentifrices may provide more significant benefits to consumers than conventional fluoride dentifrices.

## Introduction

Dental erosion is a condition of growing concern that is prevalent globally in both children and adults. Some researchers have reported that it is present in more than half of all adolescents [1–3]. The manifestation of dental erosion is primarily due to the excessive exposure of the teeth to acid-containing beverages and foods as well as gastric acid associated with gastroesophageal reflux disease (GERD) and bulimia [4, 5]. Dental erosion involves the demineralization and softening of the tooth surface which, once softened, is highly susceptible to further erosion due to forces related to abrasion and attrition, such as tongue friction and shear [6–8]. Importantly, as opposed to caries, which is a reversible condition when treated in its early stages, dental erosion is considered to be essentially irreversible, since mineral is being lost directly from the surface, rather than subsurface areas, of the affected teeth [8, 9]. There is a growing concern by dental professionals that dental erosion may represent a significant threat to the long-term health and integrity of tooth structure, necessitating intervention approaches designed to minimize any permanent damage. As a result, preventive strategies designed to protect exposed tooth surfaces against the initiation and progression of dental erosion are of high interest to the dental community [9, 10].

There are two distinct approaches to prevention of dental erosion that have been the focus of targeted research. One is to minimize the likelihood for dietary products to cause dental erosion at the source by reducing the erosive potential of acid-containing beverages and foods [8–11]; the other is to utilize oral care products to deliver a protective barrier onto exposed tooth surfaces that can serve as either a sacrificial layer or as a coating to repel erosive acid challenges [12–14]. The first of these approaches has resulted in minimal success. One issue with this approach is the likelihood of adversely modifying the taste of the acidic products when incorporating ingredients designed to minimize their erosive potential [8]. The development of oral care products that are specifically designed to help protect enamel against an increased level of erosive acid challenge, however, has been far more successful [12–15]. Oral care products are routinely used at least once a day by most individuals, and many people use them twice a day. This type of usage pattern makes oral care regimens perfectly suited to delivering product therapies that can deposit on and be retained on exposed tooth surfaces for extended periods of time and help protect these tooth surfaces against erosive acid challenges at time points beyond the initial use of the products [13, 16–18].

While many modern fluoride-containing products have been shown to provide some level of erosion benefit [12, 19], products formulated with stabilized SnF_2_ have been confirmed to be particularly effective, depositing a long-lasting barrier layer onto pellicle-coated tooth surfaces that is capable of withstanding erosive acid challenges for extended periods of time post treatment [13, 16–18, 20, 21].

A new fluoride dentifrice has recently been launched containing a combination of 1450 ppm F as sodium monofluorophosphate (SMFP), 1.5 % arginine, and calcium carbonate. The manufacturer of this dentifrice markets this product as providing enhanced enamel strengthening and superior cavity protection, due, in part, to the product’s claimed ability to neutralize plaque acids from consumption of dietary sugar [22]. Dentifrices formulated with combinations of SMFP, arginine, and calcium have been shown to be effective against caries [23], hypersensitivity [24], and dental erosion [25], although none of these studies compared the effectiveness of the arginine-based formulation versus a stabilized SnF_2_ dentifrice.

In order to determine the most appropriate oral care products for patient needs, there is an ongoing interest in the relative effectiveness of different products to help prevent dental erosion. In situ clinical and in vitro model studies have proven beneficial in measuring the relative erosion-protective effectiveness of different products. Both of the models presented here have been successfully used in the past either to predict the potential effectiveness of oral care products [16–18] or to confirm their effectiveness under human use conditions [20, 21]. The aims of this study were to compare the two marketed dentifrices for their ability to provide erosion protection benefits, using different chemistries, and to test whether these independent approaches result in differences in the erosion protection potential of the two dentifrices.

## Methods and materials

### Test products

The following dentifrices, formulated with a total of 1450 ppm F, were tested in both the in vitro and in situ studies:

- 0.454 % SnF_2_ + 0.077 % NaF (1450 ppm fluoride), marketed as Oral-B® Pro-Expert dentifrice (The Procter & Gamble Company, Gross Gerau, Germany)
- 1.1 % SMFP (1450 ppm fluoride) with 1.5 % arginine, marketed as Colgate® Maximum Cavity Protection plus Sugar Acid Neutralizer Dentifrice (Colgate-Palmolive Co., Kolombiya, Turkey)

In addition, the in vitro study included a product containing 0.454 % (1100 ppm) F as stabilized SnF_2_ (Crest® Pro-Health dentifrice, The Procter and Gamble Company, Cincinnati, OH, USA) that has been previously demonstrated in both in vitro and human in situ clinical trials to provide significant erosion protection benefits [16–18, 20, 26, 27].

### In vitro study design

**Fig. 1:**
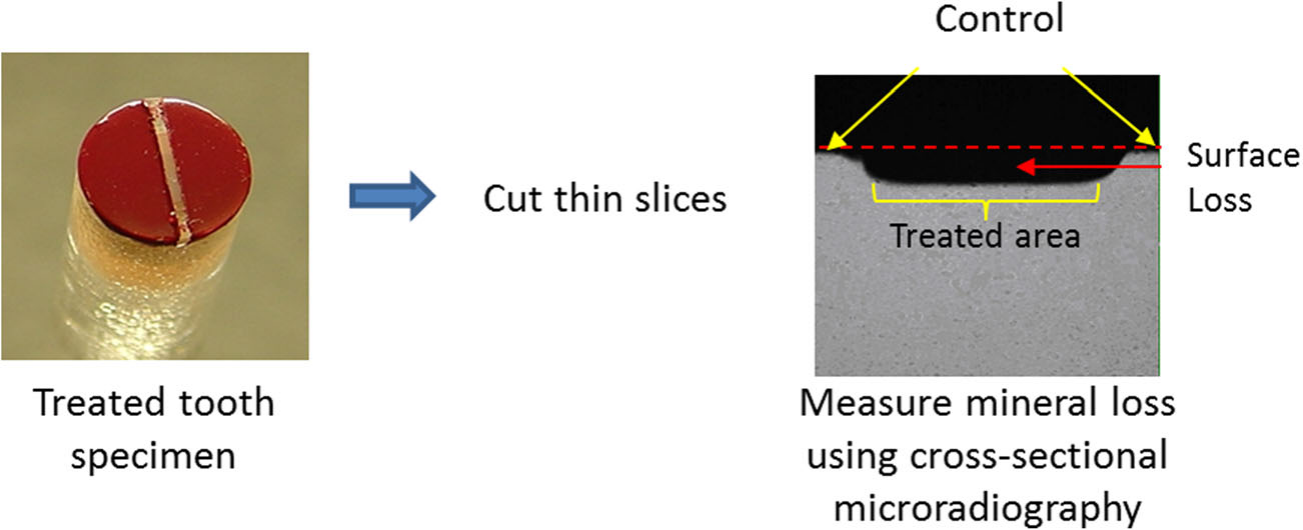
Cross-sectional micrograph of in vitro-treated specimen showing area of tooth surface loss

For the in vitro study, preparation of specimens and treatment regimen followed exactly the established protocol of Faller [17, 18]. Briefly, ground and polished cores of sound, pellicle-coated, human enamel, mounted in lucite rods, were treated in a 1:3 slurry (5 g of dentifrice/15 g of fresh, pooled human saliva) four times per day (2 min each) for a total of five treatment days. Erosive acid challenges were made by soaking each treatment group of four enamel specimens in 1 % citric acid at neat pH (approximately 2.3) for 10 min at room temperature, 1 h after each dentifrice slurry treatment. Both the product slurry treatments and the erosive acid challenges were carried out with each group of test specimens fitted to a specially designed holder and suspended into the appropriate mixture while being rotated at a controlled speed of 75 rpm to provide a constant shear. At all times specimens were not in treatment or erosive challenge, they remained in pooled, human saliva that was freshened three times each day. Assessment of surface enamel loss was made via direct measurement of cross-sectional samples taken from each specimen after completion of all treatments (Fig. 1).

#### Collection of human saliva

Approximately eight healthy volunteers were recruited to provide human saliva for this study. Fresh saliva was collected from volunteers each day of the study. Each volunteer chewed paraffin wax and expectorated the stimulated saliva into a plastic cup over a period of approximately 30 min per collection period. The saliva collected from the group of volunteers was then pooled and stored in the refrigerator at 5 °C until use.

#### Specimen collection and preparation

Enamel samples used in this study were prepared from human teeth. The teeth were obtained from local oral surgeons who collected them after removal, typically for orthodontic reasons. Teeth were individually cleaned and checked for any visible cracks or surface imperfections. Those with visible imperfections were discarded. Teeth were stored in the refrigerator in a 1 % thymol solution at 5 °C until use.

Required precautions were in place to ensure proper handling of teeth and saliva from the point of collection to their use in the laboratory study.

### In situ clinical trial

Ethical approval for the human in situ clinical study was granted by the UK National Research Ethics Service, and the study was designed and managed in compliance with the principles of good clinical practice. All participants received verbal and written information concerning the study and gave signed and witnessed consent to participate. An individual, not otherwise involved in the study, monitored the conduct of the study and the case record forms. The trial was a single-center, double-blind, randomized, supervised-usage, two-treatment, four-period crossover study. Additional periods, in this case a four-period crossover design, allow greater statistical power for detecting treatment differences in lieu of additional subjects and help to minimize the influence of individual periods. A sufficient number of participants were recruited to enroll approximately 36 participants and complete the study with at least 30 evaluable participants. Each study participant was specially fitted with an upper palatal intraoral appliance containing two enamel samples that was worn on each treatment day of the study, which was retained within their mouth using wire clasps on suitable posterior teeth.

Participants were randomly assigned at study initiation to a treatment sequence ordering their use of the two commercially available test dentifrices. Participants presented for four study periods, were randomized to treatment sequences, and received one of two dentifrices each period on test days at the clinical site. Each study period took place over a span of 2 weeks and was composed of 10 treatment days, which were conducted on weekdays only (Monday to Friday). This study was a variation of the previously published method of Hooper et al. [20].

### Prior and concomitant therapy

At the beginning of the study, participants were instructed to refrain from using any prescription or non-prescription oral care products, which were not assigned test articles throughout the duration of the study. Participants were also instructed to refrain from receiving oral prophylaxis or any other elective dental procedures throughout the duration of the study.

### In situ product use

Assigned test dentifrices were administered in the form of a slurry on each treatment day to each participant prior to the erosive challenge. Clinical site personnel prepared the dentifrice slurries for each participant by mixing 3 g of dentifrice with 10 mL of water. Participants were unaware of the product identity of their assigned dentifrice slurry, and they were instructed not to discuss the physical properties of their assigned products with other study participants or clinical site personnel. In addition, to maintain blinding, the investigator and personnel performing and recording the surface profilometry assessments had no access to the product dispensing room during treatments. At screening, the participants were provided with a non-treatment 0.32 % NaF (1450 ppm fluoride) marketed dentifrice (Crest® Decay Protection dentifrice: The Procter & Gamble Company, UK) and manual toothbrushes (Oral-B® 35 manual toothbrush: The Procter & Gamble Company, Cincinnati, OH, USA) for use at home until the follow-up visit. The participants were required to use these products in place of their normal oral care products, twice per day (morning and evening), for the duration of the study, including treatment days, weekends, and leave days.

### In situ study design

On each treatment day, participants brushed their teeth at home in their usual manner using the non-treatment dentifrice and manual toothbrush. Participants then visited the clinical trial unit where they collected their upper palatal intraoral appliance fitted with two enamel samples and placed it in their mouth. While wearing the appliance (approximately 6 h in total each day), participants swished twice a day for 60 s with their assigned dentifrice slurry under the supervision of clinic staff. The erosive challenge occurred with the appliance in their mouth. The participants sipped 25 mL of orange juice (Sainsbury’s Supermarkets Ltd., 33 Holborn, London, EC1N2HT) over a timed minute, swished it around their mouth and over the enamel specimens in order to generate shear forces, and then spit it out. The procedure was repeated 10 times per challenge so that the enamel samples were exposed to a total of 250 mL of orange juice over a 10-min period. The erosive acid challenge was carried out a total of four times on each treatment day.

Enamel samples were measured for tissue loss using a calibrated contact surface profilometer (Mitutoyo (UK) Ltd., Joule Road, Andover, Hampshire UK). Measurements were taken at baseline prior to the start of the study and at the end of treatment day 10. Fresh enamel samples were placed in the intra-oral appliance at the beginning of each treatment period.

### Preparation of enamel samples for the in situ study

Prior to the beginning of the study, enamel samples were prepared and a baseline profilometry measurement was obtained for each sample. Enamel specimens were obtained from caries-free human third molars that had been recently extracted and donated by patients aged 18 years and older, of either gender, to a licensed tissue bank (Bristol Dental School and Hospital Tooth Tissue Bank, REC Ref: 11/N1/0145). Upon donation to the tissue bank, teeth were soaked in a 20,000-ppm available chlorine solution (Haz-Tab Tablets, Guest Medical, Aylesford, Kent UK) for at least 24 h. The teeth were cleaned; the root was sectioned from the crown to enable dental pulp removal and disposal and then soaked for a subsequent 24 h in a solution containing 20,000 ppm available chlorine. The teeth were then soaked in distilled water and stored in the tissue bank in a solution containing 5000 ppm available chlorine until use.

Tooth crowns obtained from the tissue bank were sectioned into 1-mm slices using a microslice (Ultra Tec Ltd., Santa Ana, CA, USA) to produce enamel samples, which were further trimmed as needed using a high-speed handpiece and diamond bur. Each enamel sample was then placed with the test surface facing down in a mold 6 mm × 8 mm × 2 mm (width, length, and depth, respectively) and filled with epoxy resin. After 24 h, once the epoxy resin had cured, samples were removed. Each specimen was then hand polished using a standardized series of silicon carbide papers, silica powders, and polishing techniques until the surface of each specimen was sufficiently smooth and shiny, as determined through visual inspection by a trained laboratory technician.

Two baseline readings of each enamel sample were then taken using contact profilometry. Each sample was identified with a unique number on the reverse of the sample using a permanent marker and then masked with a PVC tape on either side of a 2–3-mm-wide window of enamel.

Fresh enamel samples were placed in the intra-oral appliance at the beginning of each of the four study periods. The enamel samples were retained within the appliance using wire clasps, one in the anterior of the mid-hard palate and one in the posterior region of the mid-hard palate. The appliances (containing the enamel samples) were dipped in Corsodyl® mouth rinse (0.2 % *w*/*v* chlorhexidine gluconate; GlaxoSmithKline Consumer Healthcare, Brentford, Middlesex, UK) twice daily for approximately 3 min and briefly rinsed in tap water at the start of the treatment day and upon removal from the mouth at the end of each treatment day. The appliances were removed for up to a 1-h period over lunch and also overnight until the next day. When removed at these times, the appliances were stored in a pot containing a damp cotton wool pad, moistened with water. Samples were removed from the appliances at the end of day 10 for duplicate profilometry measurements. Prior to making profilometry measurements, each appliance was disinfected by soaking in a mixture of 0.5 % chlorhexidine and 70 % aqueous ethanol for a period of at least 20 min, and then, the samples and tape were removed.

### Statistical methods

The in vitro study was analyzed for mean differences between groups using one-way analysis of variance, including the Games-Howell post hoc multiple comparison procedure since the groups demonstrated unequal variances. The primary measure of efficacy in the in situ trial was the amount of dental erosion that had occurred, measured by profilometry, at day 10. For each participant and treatment period, the average of four erosion profilometry measurements was calculated using two replicate measurements from each of two enamel sections. Since the day 10 enamel loss distribution was right-skewed, the data were transformed using the natural log function to make the distribution bell-shaped before performing between-treatment analysis that assumed normality. A general linear mixed model was used to compare treatments, and the final model included baseline, period, and treatment as fixed effects and participant as a random effect. The carryover effect was not statistically significant (*p* = 0.71) and was removed from the statistical model. From the final statistical model, estimated means on the natural log scale were back-transformed by using the exponential function (*e* ^mean^) to obtain the estimated medians of 50th percentiles on the original scale (μm), and 95 % confidence intervals (CI) were calculated. All statistical comparisons were two-sided with a significance level of 0.05. The null hypothesis tested in the human clinical study at day 10 was that the mean dental erosion was equal between the treatment differences, with the alternative hypothesis being that the mean dental erosion was not equal between the treatment differences.

## Results

In the in vitro study, specimens treated with the stabilized SnF_2_ dentifrice resulted in an average enamel surface loss (SEM) of 5.75 μm (1.03), compared to an average loss of 23.75 μm (4.27) for the SMFP/arginine dentifrice, which indicates a 75.8 % benefit in protecting the enamel against erosive acid damage, in favor of the stabilized SnF_2_ dentifrice. Compared to the positive control, the SMFP/arginine dentifrice performed at a level that was significantly less effective than this clinically proven reference product, while the stabilized SnF_2_ dentifrice provided an equivalent level of effectiveness (Table 1).

**Table 1:** In vitro efficacy results

In vitro efficacy results—treatment comparison of surface loss (μm) (*N* = 4/treatment group)				
Treatment group	Key formulation components	Surface loss (μm) ± (SEM)^a^	Statistical grouping	% Reduction vs. SMFP/arginine
Stabilized SnF_2_ control	1100 ppm F as SnF_2_	4.25 (0.95)	A	82.1
	Silica abrasive			
Stabilized SnF_2_	1100 ppm F as SnF_2_	5.75 (1.03)	A	75.8
	350 ppm F as NaF			
	Silica abrasive			
SMFP/1.5 % arginine	1450 ppm F as SMFP	23.75 (4.27)	B	–
	1.5 % arginine			
	CaCO_3_ abrasive			

^a^Means with different letter designations are significantly different (*p* < 0.05)

In the in situ evaluation, 34 participants (mean age 44.6 years) were randomized to treatment; 33 participants completed the final study visit. The baseline profilometry measurements of the surface of specimens included in the in situ study were nearly zero, with means (SE) of −0.090 (0.0095) and −0.069 (0.0095) for the stabilized SnF_2_ and SMFP/arginine dentifrices, respectively. Due to a low standard error of the mean, a statistically significant difference (*p* < 0.05) was observed between the two test dentifrices at baseline. To address this in the statistical model for the day 10 treatment comparisons, baseline was used as a covariate to obtain adjusted means for each test dentifrice. Despite the statistically significant baseline mean difference of 0.021, the day 10 treatment mean difference was nearly 55 times larger.

**Fig. 2:**
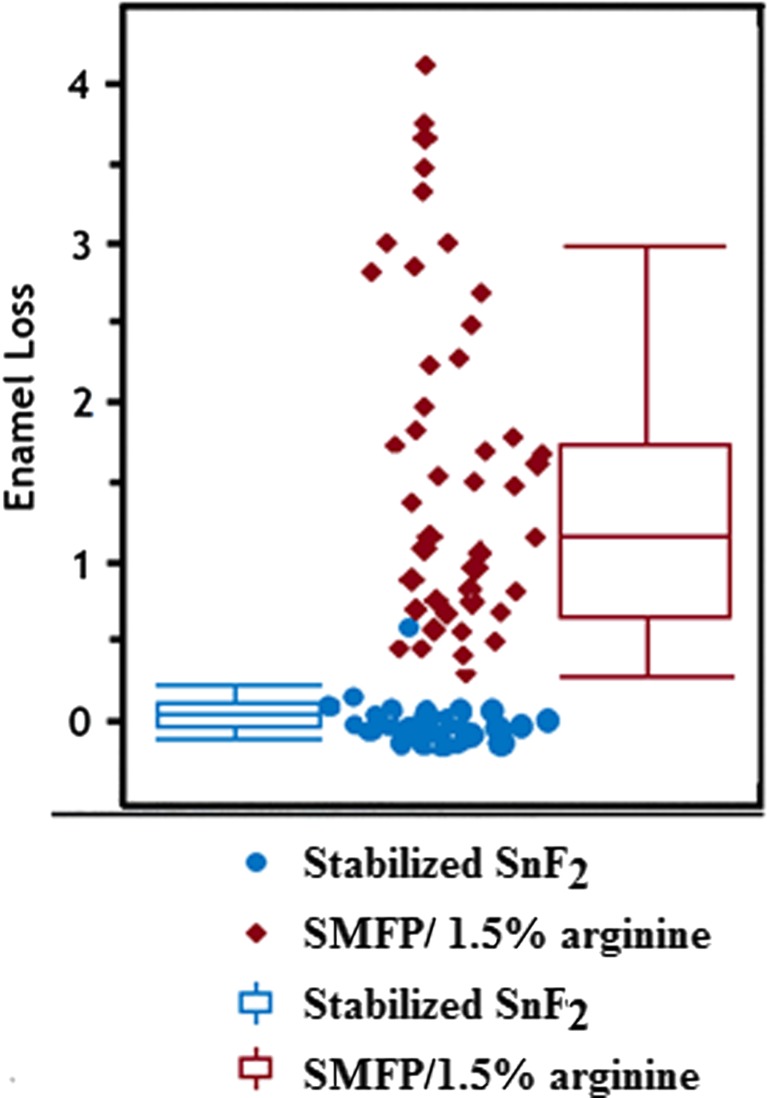
Distribution box plot—enamel loss data (μm) at day 10

At day 10, the stabilized SnF_2_ dentifrice demonstrated a statistically significant (*p* < 0.0001) 93.9 % better protection against erosion versus the SMFP/arginine dentifrice with estimated enamel loss medians (CI) of 0.075 μm (0.060, 0.093) for the stabilized SnF_2_ dentifrice and 1.226 μm (0.980, 1.532) for the SMFP/arginine dentifrice (Table 2). On the natural log scale, the estimated means from the statistical model were calculated for the dentifrices, and the estimated medians in micrometers, above, were calculated by applying the exponential function (Table 2). The SnF_2_ dentifrice resulted in more than 10 times better enamel protection versus the SMFP/arginine dentifrice. A distribution box plot of enamel loss by treatment (Fig. 2) verifies distinct differences in performance between the two test dentifrices at day 10. Both dentifrices were well tolerated. There were no adverse events in the study.

**Table 2:** Day 10 in situ efficacy results

In situ efficacy results—treatment comparison of profilometry levels (μm) general linear mixed model evaluable participants (*N* = 34)				
Visit/treatment	Original scale in μm estimated median (SE)^a^	95 % Confidence interval of the estimated mean	Natural log scale mean (SE)	% Reduction vs. SMFP/arginine (*p* value)^b^
10 days post baseline (participant variance = 0.0264, residual variance = 0.7869)				
Stabilized SnF_2_	0.0747 (0.008)	(0.060, 0.093)	−2.5946 (0.1116)	93.9 % (*p* < 0.0001)
SMFP/1.5 % arginine	1.2255 (0.138)	(0.980, 1.532)	0.2033 (0.1125)	

^a^Estimated medians in micrometer were obtained by using the exponential function on the means from the natural logarithm scale (*e* ^mean^)

^b^Percent reduction was calculated using back-transformed means as 100 % (SMFP/1.5 % arginine − Stabilized SnF_2_) / SMFP/1.5 % arginine. Two-sided *p* values for testing the mean difference between treatments were provided

## Discussion

The use of in vitro models designed to simulate conditions of excessive erosive acid ingestion has proven to be valuable for predicting the likely erosion protecting performance of oral care products when tested under conditions of actual human use. One of the primary benefits to the use of in vitro models is the ability to control conditions in such a way that all treatments are done in an essentially identical manner. Standardization in the in vitro models also generally results in lower variability that is helpful for predicting ultimate performance in human clinical studies.

Stabilized stannous fluoride dentifrices have been comprehensively evaluated against a broad range of marketed products in well-credentialed, standardized, and validated in vitro and in situ model studies, with the results of these studies published in several peer-reviewed journals [16–18, 20, 21, 27, 29]. The credentialed in vitro model has proven to be a strong predictor of in situ performance and, for that reason, has been included in this report where the same two products included in the in situ study were tested, along with a positive control, 1100 ppm F as SnF_2_, dentifrice.

In the present paper, the results predicted by the in vitro erosion cycling model were subsequently confirmed in the human in situ clinical trial, with both studies demonstrating that the stabilized SnF_2_ dentifrice provided high percentage differences in performance compared to the SMFP/arginine dentifrice. The erosion cycling model provides a useful simulation of the process of dental erosion, beginning with sound, pellicle-coated human enamel, subjecting the pellicle-coated enamel to erosive acid challenges for controlled periods of time that mimic human use of erosive acid beverages, bathing in pooled, human saliva to allow remineralization processes to occur, and incorporation of shear forces capable of removing softened enamel from the treated surfaces. In the in situ model, specimens are exposed to natural saliva flow, enabling the deposition of a naturally formed pellicle on the enamel specimens. Both models reported here are well accepted to assess the ability of each test product to protect sound, treated surfaces against tooth surface loss due to erosive acid challenge, which was the objective of this research. Neither model includes direct, physical brushing (i.e., abrasion) of the enamel specimens as that was not the objective of these studies. Other research groups have made attempts to incorporate abrasion into either in vitro [30–34] or in situ [35, 36] models. At times, these models show similar rank-ordered results when comparing the erosion only versus erosion–abrasion results [32, 34, 35]. In other studies, results were different when comparing erosion to erosion–abrasion outcomes [30, 31, 33]. Thus, to ensure the single-variable objective of this research, brushing was not included. Results should be viewed in this context when extrapolating them to the oral environment. Further evolution of these models, to assess both the chemical erosion-preventive effects of the paste in addition to the effects of a mechanical intervention, should be a topic for future research.

One issue faced by researchers conducting human erosion studies is the safety of the subjects’ natural teeth against the erosive conditions prescribed by the study protocol. The study design used in the current evaluation is considered to be completely safe for the subjects’ natural teeth, as it is not expected to cause any significant loss of enamel. Subjects are evaluated prior to the study to ensure that they show minimal evidence of tooth wear. The protocol required that if any subject lost 20 μm or more of the test enamel, the subject would be pulled immediately from the study. As the results demonstrate (Table 2), the total amount of loss on the specimens treated with the less effective product resulted in only a few microns of surface lost over the duration of the study.

Results of the current study are in line with previously published results that made use of the same in vitro model as well as the same, or similar, in situ models. Evaluations of a marketed, stabilized SnF_2_ dentifrice using the in vitro erosion cycling model demonstrated results favoring the stabilized SnF_2_ dentifrice by 65–86 % over other marketed products tested [16–18]. Published in situ evaluations have resulted in 56–87 % benefits in erosion prevention relative to comparator products [20, 21, 27], depending on the study design.

The ability of different formulations to deposit onto treated surfaces and strengthen those surfaces against erosive acids, thereby minimizing the potential for irreversible tooth surface loss, is an important factor to consider when assessing the potential effectiveness of oral care products. Products formulated with either stabilized SnF_2_ or arginine, calcium, and SMFP are claimed to be effective against caries as well as hypersensitivity, with erosion being an etiological factor for dentin hypersensitivity as an outcome of the loss of the enamel and or cementum and the loss of the outermost layers of mineral that coat the dentin tubules [28, 29]. Although both types of formulations included in the current study make claims of sensitivity benefits as well as erosion protection, they do so via different chemistries.

With respect to the ability of arginine, calcium, and SMFP to reduce sensitivity, the manufacturer claims the “technology works by physically sealing dentin tubules with a plug that contains arginine, calcium carbonate, and phosphate” and it is this mass that reduces acid solubility [37]. Thus, the chemistry is essentially a calcium phosphate deposit. It is likely that this same type of surface deposit would also be responsible for any erosion protection benefit delivered to enamel, since both hypersensitivity and dental erosion are eventual outcomes that may result from similar types of acid challenges. Stabilized SnF_2_ dentifrices have been demonstrated to physically seal exposed dentin tubules via the chemical precipitation of a stannous-rich, acid-resistant smear layer [38, 39]. Deposition of an acid-resistant smear layer is likely the key mechanism for protection of dentin against both sensitivity and dental erosion. Smear layers act in a sacrificial manner, preferentially dissolving prior to the acid attacking the dentin itself, resulting in neutralization of the acid challenge. Studies by Rees et al. [40] and Pinto et al. [41] assessed the impact of acid-containing beverages on smear layer removal, finding that more aggressive beverages (measured in terms of pH, acid type, acid content, and titratable acidity) resulted in faster removal of the smear layer and more rapid exposure of occluded tubules. The overall aggressiveness of an acid challenge against dentin can be reduced by deposition of a more acid-resistant smear layer. Studies by White et al. [42] reported that dentin treated in vitro with a stabilized SnF_2_ dentifrice resulted in enhanced resistance to acid dissolution and tubule exposure. In addition to its ability to deposit on dentin surfaces, SnF_2_ has been shown to deposit onto hydroxyapatite surfaces [43], and stabilized SnF_2_ dentifrices have been shown to deposit a protective, acid-resistant, stannous-containing barrier layer onto both sound enamel and pellicle-coated enamel surfaces [13, 14]. Importantly, the stannous-containing barrier layer has been demonstrated to remain on the pellicle-coated tooth surfaces for at least several hours after treatment [13].

The barrier layer formed after treatment with stannous-containing products is most likely composed of either stannous fluorophosphate or stannous oxide compounds [19], either of which would be expected to provide significantly higher resistance to erosive acids compared to a precipitate composed primarily of calcium and phosphate, which is likely the type of deposit delivered from dentifrices containing SMFP, arginine, and calcium [16, 26, 30]. Importantly, the in situ human clinical trial demonstrated greater than 10 times better enamel protection associated with the deposition of an acid-resistant, stannous-containing barrier layer.

Results from the current studies support the superiority of stabilized SnF_2_ dentifrices for protecting human teeth against the initiation and progression of dental erosion.
